# Tuning the Solid-State Hydrogen Release of Ammonia Borane by Entrapping the Intermediates: The Role of High-Boiling-Point Amines

**DOI:** 10.3390/molecules30204057

**Published:** 2025-10-11

**Authors:** Mattia Bartoli, Giuseppe Ferraro, Marco Etzi, Stefania Lettieri, Candido Fabrizio Pirri, Sergio Bocchini

**Affiliations:** 1 Center for Sustainable Future Technologies—CSFT@POLITO, Via Livorno 60, 10144 Torino, Italy; marco.etzi@iit.it (M.E.); fabrizio.pirri@polito.it (C.F.P.); sergio.bocchini@polito.it (S.B.); 2 Consorzio Interuniversitario Nazionale per la Scienza e Tecnologia dei Materiali (INSTM), Via G. Giusti 9, 50121 Florence, Italy; 3 Department of Applied Science and Technology, Politecnico di Torino, C.so Duca degli Abruzzi 24, 10129 Turin, Italy; giuseppe.ferraro@polito.it (G.F.); stefania.lettieri@polito.it (S.L.)

**Keywords:** ammonia borane, hydrogen storage, reactivity, chemical bonded hydrogen

## Abstract

Ammonia borane is a promising hydrogen storage material due to its high hydrogen content, but its use as hydrogen carrier under thermal stimuli involves the production of several byproducts, such as borazine, reducing hydrogen purity and the overall efficiency. This work is focused on the use of high-boiling-point amines to modulate ammonia borane decomposition, aiming to enhance hydrogen release and suppress volatile N_x_B_y_ species. Kissinger’s equation kinetics revealed that amines significantly influence the decomposition mechanism, and TGA-IR investigation showed a maximum of 2.4 wt.% of pure hydrogen release in the presence of triphenyl amine. Furthermore, the experimental data herein discussed, together with a computational study of activation energies, allowed us to derive a detailed mechanism that leads to a foundation for further advancement in the exploitation of ammonia borane as a hydrogen carrier, suggesting that the formation of linear species is anchored to amine over the release of borazine and production of poly borazine-like species.

## 1. Introduction

Nowadays, the critical issues related to hydrogen mainly concern its distribution into energetic grids and its safe storage [1]. Actually, all high hydrogen volumes are stored as liquid hydrogen in expensive insulated vessels that already undergo product losses due to evaporation [2]. Accordingly, the use of liquid hydrogen is limited to those applications that require high volume and pure gas such as chemical, metallurgic, and aerospace industries. Another quite rare approach used to store hydrogen is as compressed gas. Nevertheless, compressed hydrogen can form combustible or explosive mixtures with air over a wide range of concentrations with high diffusivity through several metal alloys. This can lead to embrittlement and crack formation with possible hydrogen leaks [3] and can represent the first limitation to its use in the automotive sector for hydrogen-based vehicles [4,5]. The other issue in storing hydrogen for energetic applications is related to its energy content, which under standard temperature and pressure conditions is lower than liquid fuels [6]. As has clearly emerged, the combination of these issues drastically limited the use of hydrogen technologies in mobility, preventing major automotive manufacturers from marketing vehicles based on technologies, which are using hydrogen as a fuel, due to their safety limitations [7]. Several approaches have been developed for storing and using hydrogen under safe conditions with a high energy density [8]. Among the available solutions, chemical storage represents the best performing route with which to achieve both high volumetric and gravimetric energy density [9]. Nevertheless, traditional approaches, including the cracking or reformation of small molecules (i.e., methane, ammonia) are energetically intense and hard to pair with technologies for mobility [10,11]. Alternatively, the utilization of hydrides (i.e., LiBH_4_ [12], MgH_2_ [13]) is quite promising, as they can reach a volumetric energetic density of 17.6 MJ/L [14]. Nevertheless, these species are highly sensible to water and air and are poorly regenerable. Ammonia borane (AB) represents a more stable alternative hydrogen carrier with a theoretical gravimetric hydrogen storage capacity of up to a remarkable 19.6 wt.% and relatively good stability in water and air. As reported by Geanangel et al. [15], AB melts at around 115 °C, while it starts to release H_2_ at around 85 °C [16]. Meanwhile, AB water solutions are stable at room temperature in the absence of catalysts [17] below 40 °C up to a concentration of 8 M [18]. Nevertheless, AB has shown a very complex reaction mechanism during hydrogen release under thermal stimuli [19] with the release of borazane species [20,21,22], while catalytic mediated hydrolysis is simpler but less energy-efficient [23,24,25]. This is a considerable drawback, as the high purity of hydrogen required for applications such as fuel cells limits its use. Several studies [24,26,27,28] have explored the utilization of the confinement approach or utilization of catalysts to tune and modulate AB reaction pathways by depleting the release of borazine [29] and partial dehydrogenated polymeric N_x_B_y_ species [30].

In this work, we first explore the utilization of a high-boiling-point amine as a modulating agent for tuning AB reaction pathways during thermal-induced decomposition for hydrogen release. The reason behind this approach is the exploitation of the catalytic effect of amine towards the formation of adducts able to confine into solid state the major degradative pathways for hydrogen release. Importantly, coordination with high-boiling-point amines also limits the thermal volatilization losses of AB, which tends to evaporate at relatively low temperatures.

## 2. Results and Discussion

### 2.1. Evaluation of Activation Energy of Thermal Degradation of AB in Presence of High-Boiling-Point Amines Through Non-Isothermal Kissinger Equation

**Figure 1:**
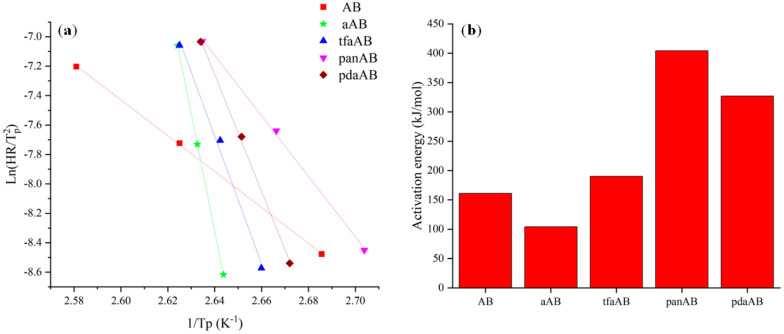
Output of DSC elaboration using Kissinger equation, reporting (**a**) Kissinger plot and (**b**) activation energies of AB, aAB, tfaAB, panAB, and pdaAB.

The thermal reaction pathway of AB is quite complex, and it involves the release of a molecule of H_2_ as the first stage. The activation energy of this first degradative step [31] can be assessed through DSC analysis using the Kissinger equation [32]:

(1) $$\ln\left(\frac{HR}{T_{p}^{2}}\right)=\frac{{-E}_{a}}{{RT}_{p}}+\ln\left(\frac{Z}{{RE}_{a}}\right)$$

where *T*_p_ is the peak temperature, HR is the heating rate, E_a_ is the activation energy, Z is a kinetic constant, and R is the ideal gas constant. The Kissinger equation is used to evaluate the activation energy of AB dehydrogenation through the plot of ln(*α*/*T*_p_^2^) versus 1/*T*_p_ using a linear fit, as shown in Figure 1.

As reported in Figure 1a, AB was characterized by an E_a_ of 160 kJ mol^−1^, close to the one reported by Gutowska et al. [33] of up to 161 kJ mol^−1^. aAB showed a decrement in E_a_ down to 104 kJ mol^−1^, while tfaAB showed an increment of 19% in E_a_ compared with AB, reaching up to 191 kJ mol^−1^. A further increment in E_a_ was observed for both panAB and pdaAB that reached up to 404 and 327 kJ mol^−1^, respectively, suggesting that other factors, beyond amine-induced destabilization, are involved, as reported by Kim et al. [34], for metal-free Ab degradation in the presence of ethers.

The E_a_ data together with the different slopes of Kissinger’s plot (Figure 1a) suggest that the reaction mechanism shifts towards different pathways moving from aAB towards highly hindered or multifunctional amine.

### 2.2. Solid-State Thermal Degradation of AB in Presence of High-Boiling-Point Amines

**Figure 2:**
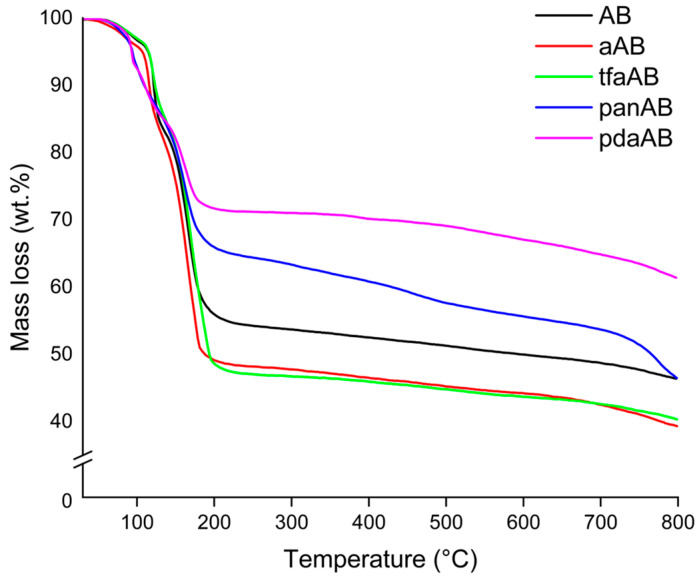
Thermograms of AB, aAB, tfaAB, panAB, and pdaAB, collected using heating rate of 10 °C/min.

**Figure 3:**
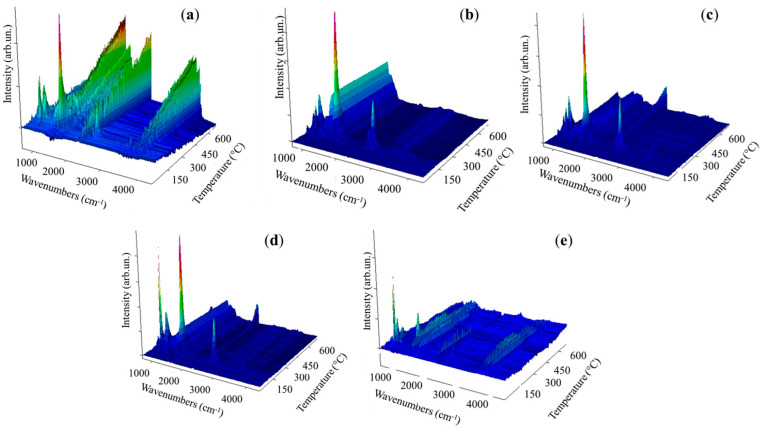
IR spectra collected during analysis of AB using heating rate of 10 °C/min of (**a**) AB, (**b**) aAB, (**c**) tfaAB, (**d**) panAB, and (**e**) pdaAB.

The amine was selected primarily by considering the boiling point being higher than first degradative stage of AB in order to entrap at least the very first intermediates produced during the thermal degradation of AB. The chemical features of the high-boiling-point amine were selected to investigate specific contributions, such as steric hindrance and chemical functionalities. The solid-state thermal degradative behaviour of AB in the presence of high-boiling-point amines was investigated through TGA-IR analysis, as reported in the thermogram of Figure 2 and IR spectra of Figure 3, and the data were reported in Table 1.

According to TGA-IR analysis (Figure 2 and Figure 3 and Table 2), AB released pure H_2_ until a temperature of 31 °C was reached with a consequent mass loss of only 0.2 wt.%. Above this temperature, ammonia was released, as proved by the increases to ν_NH_ 965 cm^−1^ and 930 cm^−1^ [35], and also diborane signals τ_HBH_ 2584–2523 cm^−1^, ν_BH_ 2361–2329 cm^−1^, δ_BH_ 1387 cm^−1^ were observed [36], suggesting a chemical pathway involving the thermolysis of the N-B bond of AB. At 86 °C, two signals were observed at 1392 cm^−1^ and 1334 cm^−1^ related to the ν_BN_ of NH_2_BH_2_ residues, as discussed by Gerry et al. [37], while above 100 °C, borazine was formed, as confirmed by the bands in the 1470–1460 cm^−1^ and 2741–2396 cm^−1^ regions [38]. Borazine release reached a maximum at 162 °C, and the residue of AB at 800 °C was up to 43.1 wt.%. aAB began decomposition at t 112 °C, showing a first degradative stage at 119 °C with a mass loss of 21.9 wt.%, a second one at 167 °C with a further decreased 7.4 wt.%%, and a third one at 172 °C with an additional weight loss of 13.1 wt.%, showing a final residue at 800 °C of 40.2%. This value was lower than that of AB, suggesting a more extensive volatilization in accordance with the enhanced pure hydrogen release of 2.4 wt.%. A similar be haviour was observed for tfaAB, while panAB and pdaAB showed lower initial decomposition temperature but higher final residues up to 43.8 and 58.2 wt.%, respectively. The TGA analysis proved that AB undergoes two main degradation stages with moderate hydrogen and volatile release, while aAB and tfaAB showed three distinct steps, resulting in greater cumulative mass loss and higher hydrogen production. panAB and pdaAB decomposed at lower temperatures, highlighting the influence of amine substituents on the thermal stability and volatilization behaviour of AB derivatives.

**Figure 4:**
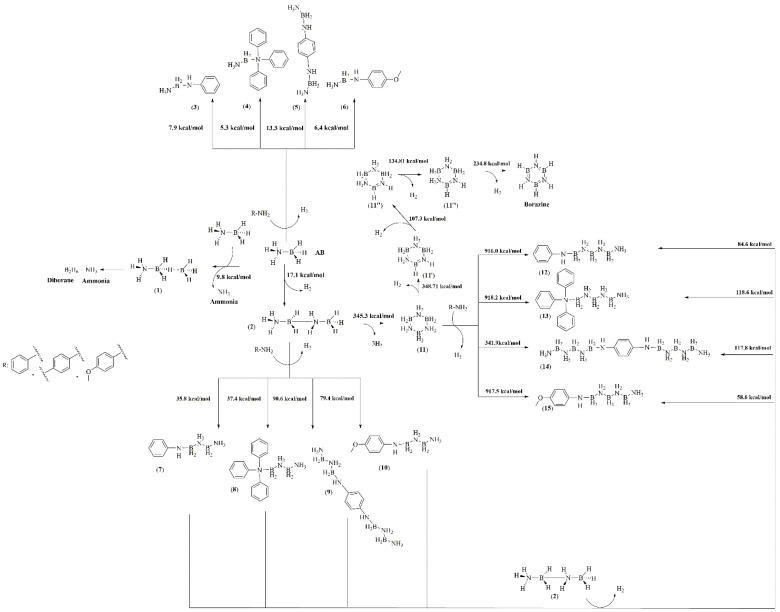
Mechanism of thermal-induced solid-state degradation of AB in the presence of high-boiling-point amines.

aAB and tfaAB showed a release of ammonia with temperatures quite close to 85 and 83 °C, respectively, while panAB and pdaAb showed a significant decrement reaching up to 70 and 71 °C. The production of ammonia was reasonably correlated with the pKa of amine considering that aniline and triphenylamine showed a pKa of 4.1 and 3.04, respectively, while benzene-1,4-diamine and 4-methoxyaniline reached 4.5 and 5.3. We hypothesize that the dissociation of N-B at low temperature was mainly promoted by the interaction between more acidic nitrogen atoms and a boron site, as reported by Heldebrant et al. [39] through the use of ammonium chloride. The reduction in ammonia release also induced a greater pure H_2_ release that reached up to 2.4 wt.% from aAB and 1.5 wt.% for tfaAB, while the other species reached around 1 wt.%. This affected the first appreciable degradative stage occurring at around 112–115 °C for both aAB and tfaAB, while panAB and pdaAb again showed a reduction in the temperature threshold down to 92 °C for the release of diamino borane mixed with borane and ammonia. Interestingly, the active mode of borazine was not detected here but bands due to ammonia and the ν_BN_ at 2591–2523 cm^−1^ were, together with the ν_NH_ (3510–3320 cm^−1^) and ν_CC_ (1622 cm^−1^) of the aromatic ring of aniline in vapour phase as reported by Mukherjee et al. [40]. In considering the boiling point of aniline reported in Table 1, the presence of aniline in vapour phase was reasonable due to the formation of aniline AB adduct, in which a NH_x_ terminal was replace with the aniline itself. A similar signal pattern was also observed for tfaAB, panAB, and pdaAB. Similarly, the second degradative stage occurring at 160 °C for all samples contains the same signal of amine bonded to N_x_B_y_ chains with a ratio between the ν_CC_ and ν_NB_ double bond, which peaked at 1461 cm^−1^ and changed with a significant increment in the second one. This suggests the growth of N_x_B_y_ on amine centres starting the release borazine only above 241 °C together with the detection of ν_NH_ at 3300 cm^−1^ due to small linear N_x_B_y_ [41]. Furthermore, large bands at 962, 934, and 913 cm^−1^ were detected in all IR spectra, suggesting the presence of nitrogen termination of amine-bonded species, such as those reported in Figure 4. The final residues ranging from 36.5% for tfaAB to 58.2% for pdaAB emphasize the difference between derivatives that underwent improved volatilization with H_2_-enhanced release and those that preferentially stabilize into cross-linked polymeric N_x_B_y_ networks, thereby retaining a larger condensed fraction.

**Figure 5:**
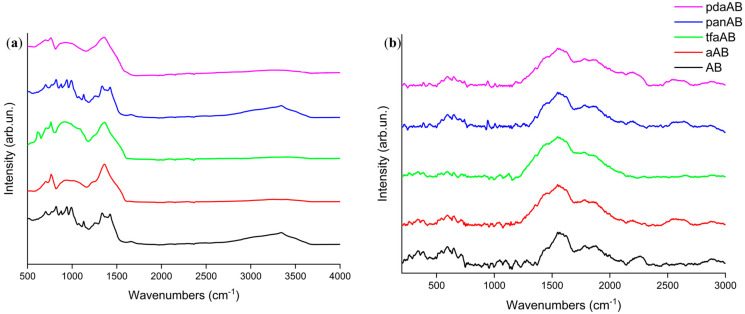
Analysis of residues recovered from thermal degradation of AB in presence of high-boiling-point amines through (**a**) FT-IR (ATR mode) and (**b**) Raman spectroscopy.

The complexity analysis of AB reaction pathways, also with amine as an additive, involves not only spectroscopical analysis to prove the advantage of a chemical pathway over another but also computational support, which allows us to calculate the activation energies of each step reported in Figure 4. AB reaction with amine was the energetically favourable pathway with the formation of single adducts (**3**–**6**) compared with the release of ammonia, which requires an activation energy of 9.8 kcal/mol. Nevertheless, these species formed at low temperatures cannot be detected in vapour phase due to their high boiling point. By increasing the temperature, the formation of adducts **7**–**10** was considerably favourable compared with the production of borazine, which requires an activation energy over 300 kcal/mol, while that for the others range from 35.9 up to 90.4 kcal/mol. Interestingly, the formation of **7** and **8** showed a lower Ea than the other intermediates, while the conversion among adducts **11** to **12**–**15** was highly disadvantageous with activation far higher than the one required for the dehydrogenation pathway leading to borazine. This suggests that inhibition of borazine release followed a pathway involving the reaction of high-boiling-point amine with AB in the early stage of the thermal degradation process. Additionally, the growth of N_x_B_y_ chains over the amine centres forming **12**–**15** with an average activation energy of 80 kcal/mol for **12** and **15** and above 100 kcal/mol for **13** and **14**. This was likely due to the elevated steric hindrance of both triphenylamine and benzene-1,4-diamine adducts. Furthermore, after these intermediate compounds were formed at a temperature above 170 °C (Table 2), only borazine was released, suggesting the rapid conversion of **12**–**15** to borazine. As shown by TGA-IR (Figure 3), a part of the high-boiling-point amine was dragged up in vapour phase forming several terminal N_x_B_y_ adducts. The fate of the one entrapped in solid was investigated through both FT-IR and Raman spectroscopy, as reported in Figure 5.

**Figure 6:**
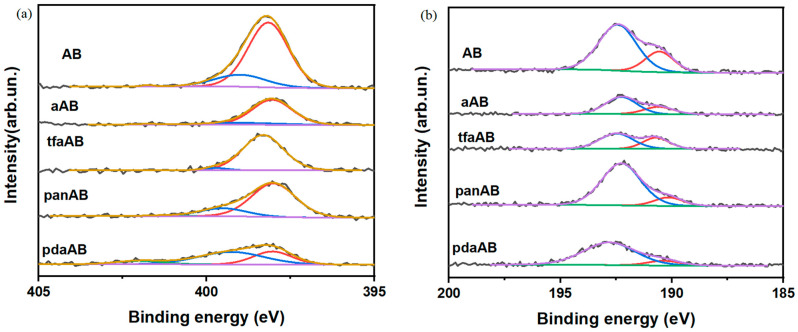
XPS spectra of (**a**) N 1s and (**b**) B 1s of residues recovered from thermal degradation of AB, aAB, tfaAB, panAB, and pdaAB.

As shown in Figure 5b, the Raman spectra of all residues recovered after TGA analysis showed broad and very weak enveloped peaks at around 1417 and 1800 cm^−1^. The Raman spectra patterns suggest a close relation with the asymmetrical ν_BN_ centred at 1370 cm^−1^, as reported by Parker et al. [42]. It is attributed to the unsaturated N-B bonds, and it can be attributed to pyrolyzed polymeric species, as reported by Impens et al. [43]. Accordingly, the residue recovered after the TGA-IR analysis appeared to be composed of a condensed borazine and highly amorphous borazine-like structure. The absence of well-defined D and G peaks was attributed to the graphitic carbon [44], suggesting the absence of condensed carbon-based aromatic domains supporting the release of high-boiling-point amine in vapour phase. As shown in Figure 5a, FT-IR of the solid residue showed a relevant envelope of bands between 1500 and 1200 cm^−1^ [45] due to both the ω_N-H_ (1410 cm^−1^) and bending mode of condensed N_x_B_y_. A quite good intensity of ν_N-BH_ at 758 cm^−1^ is also present [46]. Interestingly, we did not observe any signal in either IR or Raman spectra due to the carbon reaching derived structures supporting the removal of carbon containing amine during the degradation steps. Nevertheless, the complexity of solid residue required additional investigation to evaluate the bond speciation of both nitrogen and boron through XPS, as reported in Figure 6 and summarized in Table 2.

**Table 2:** XPS analysis output of chemical functionalities from N 1s- and B 1s-fitted spectra.

Specie	Nitrogen (%)			Boron (%)	
	N-B	NH/NH_2_-B	NH_3_-B	NB	B-O
AB	80.7	18.8	0.5	25.6	74.4
aAB	85.8	12.2	1.9	29.8	70.2
tfaAB	96.4	3.6	0.0	36.4	63.6
panAB	79.1	19.8	1.2	12.9	87.1
pdaAB	34.0	54.9	11.1	11.6	88.4

As shown in Figure 6a, the N 1s of the solid recovered after the thermolysis of AB and AB mixed with high-boiling-point amine showed three components at 398.2 and 399.1 and a small component at 401.7 eV due to N-B [29] and hydrogenated N-B species [47]. Meanwhile, B 1s showed two components at 190.5 and 192.2 eV, respectively, due to B-N and B-O species [48] formation after exposition to the air of the residues. As reported by Castilla-Martinez et al. [29], the N_x_B_y_ species derived from spent AB showed two different and well-defined behaviours based on structures that can span from borazine-like polymers or linear ones that are more and less resistant to the oxidation. As reported in Table 2, all samples showed a very high oxidation degree of boron above 86%, suggesting the presence of linear species with a reduction in oxidation for the residue of tfaAB down to 63.6%. This was likely due to the steric hindrance of the triphenylamine that promotes the formation of polyborazine, which was free to condense once the amine was fully depleted from the system with improved hydrogen release, as shown also by the residue of only 36.5 wt.% (Table 2) recovered after thermal degradation, significantly lower than that of the other mixtures. The N 1s spectra suggest the massive formation of N_x_B_y_ species with various degrees of dehydrogenation with tfaAB residue, reaching a quasi-fully dehydrogenated state with a 96.4 of BN species, while pdaAB was considerably less efficient. This was likely due to the weak interaction occurring between the ethoxy function of 4-methoxyaniline that can stabilize the boron, preventing the addition of further amines, as reported by Chabanne et al. [49]. Furthermore, the formation of linear dehydrogenated N_x_B_y_ species was in good agreement with computational analysis, which shows that the growth on amine centres was favourable upon cyclization. The absence of any evidence of C-N and B-N bonds also supports the release of amine during the thermolysis of AB without the formation of any carbon-containing species.

## 3. Materials and Methods

### 3.1. Materials

All solvents (THF > 90%) and reagents (AB purity > 95%, Aniline purity > 98%, triphenylamine purity > 98%, benzene-1,4-diamine purity > 98%, 4-methoxyaniline purity > 98%,) were purchased from Merk-Sigma-Aldrich (Darmstadt, Germany) and were used as received without any further purification.

### 3.2. Preparation of High-Boiling-Point Amine/AB Mixtures

High Boiling point amines were mixed with AB by dissolving 50 mg of high-boiling-point amines, as reported in Table 3, in 10 mL of tetrahydrofuran with an AB/amine molar ratio of ratio 10:1 in inert atmosphere using a Schlenk line system. The THF was firstly removed using a nitrogen flux at room temperature, and the solid mixtures were dried under vacuum at 40 °C overnight prior to analysis.

Table 3 summarizes the amines used, together with their acronyms and major physio-chemical properties.

### 3.3. Physicochemical Characterization of High-Boiling-Point Amine/AB Mixtures

TGA-IR analyses were carried out using a Thermo-gravimetric Analyzer NETZSCH TG 209 F1 (NETZSCH, Selb, Germany) connected to a transfer line heated at 230 °C with an IR Bruker TENSOR II (Billerica, MA, USA) equipped with an IR gas cell heated at 200 °C. The tests were performed by heating about 3 mg of the sample from 30 to 350 °C with a rate of 10 °C/min in alumina pans under a nitrogen flux of 40 mL/min.

Fourier transform IR in attenuated total reflection (FT-IR ATR mode) was conducted using a Bruker Tensor II Fourier transform spectrophotometer. The spectra were acquired by accumulating 64 scans in the range from 500 up to 4000 cm^−1^ with a resolution of 2 cm^−1^.

Differential scanning calorimetry (DSC) was performed using a NETZSCH DSC 204 F1 Phoenix instrument, equipped with a low-temperature probe. The experiments were carried out between −70 and 180 °C with a scan rate of 10 °C/min in N_2_ atmosphere (20 mL/min). The activation energy of the dehydrogenation process of the high-boiling-point amine/AB mixtures was calculated using the Kissinger equation with data collected through DSC analyses using a NETZSCH DSC 204 F1. Analyses were carried out under dry N_2_ gas (70 mL/min) by weighing 3 mg of each sample and with heating rates of 10, 5, and 2 °C min^−1^.

The solid residues recovered from TGA-IR measurements were investigated using X-ray photoelectron spectroscopy (XPS) with a PHI 5000 VersaProbe Physical Electronics (Chanhassen, MN, USA) scanning X-ray photoelectron spectrometer equipped with a monochromatic Al K-alpha X-ray source with 1486.6 eV of energy, a 15 kV voltage, and 1 mA anode current.

The solid residues recovered from TGA-IR measurements were investigated through Raman analysis using a LabRAM Soleil (Horiba, Palaiseau, France) equipped with a 785 nm laser line and 100× magnification in the range from 150 to 1000 cm^−1^.

### 3.4. Calculation of Formation Energy of Intermediates Formed During Solid-State Thermal Degradative of AB in Presence of High-Boiling-Point Amines

The activation energies of intermediates formed during the solid-state thermal degradation of AB in the presence of high-boiling-point amines were calculated using Hyperchem 8.0.1. The optimization was performed with the semiempirical Parametric Method 3 (PM3) [50] using an optimization algorithm based on the conjugate gradient (Polak–Ribiere) with a set limit of energy convergence of 0.01 kcal/Åmol [51] using a box containing 20 molecules of AB and 2 of amine.

## 4. Conclusions

This study investigated the modulation of AB thermal decomposition using high-boiling-point amines as tuning agents to enhance hydrogen release, minimizing byproduct formation, particularly aiming to reduce the release of borazine. Kissinger equation-based calculations revealed that the presence of different amines significantly altered the activation energy and decomposition pathways of AB, with aAB and tfaAB reducing the activation energy of hydrogen release, suggesting the catalytic destabilization of AB without the degradation of the N-B bond. Conversely, panAB and pdaAB increased the activation energy, suggesting the activation of alternative pathways with the formation of amine-anchored adducts. The combination of the analytical investigation of both gases released during thermal degradation and solid residues allowed the development of a well-defined mechanism, which was further supported by computational analysis. This approach led to an increased release of pure hydrogen (up to 2.4 wt.%), a reduced formation of volatile N_x_B_y_ species such as borazine, and a suppression of thermal volatilization losses of ammonia borane. These outcomes prove that the thermal decomposition of AB can be effectively modulated through rational selection of amines with suitable boiling points, pKa, and steric profiles improve the quality and quantity of hydrogen released. The findings provide a promising route toward safer, more efficient chemical hydrogen storage system for applications where purity and controlled release are key requirements, particularly in portable power sources and fuel cell technologies.

## Figures and Tables

**Table 1:** TGA analysis of main output of solid-state thermal degradative behaviour of AB in presence of high-boiling-point amines.

Species	T_onset_ (°C)	T_max1_ (°C)	Residue @ T_max1_ (%)	T_max2_ (°C)	Residue @ T_max2_ (%)	T_max3_ (°C)	Residue @ T_max3_ (%)	Residue @ 800 °C (%)	H_2_ Solo Production (wt.%) ^a^
AB	105	106	93.4	162	75.1	Not observed	Not observed	43.1	0.2
aAB	112	119	79.1	167	71.7	172	58.3	40.2	2.4
tfaAB	115	120	84.7	168	74.1	180	62.7	36.5	1.5
panAB	92.1	104	86.7	162	79.5	Not observed	Not observed	43.8	1.0
pdaAB	92.0	111	87.9	162	73.1	Not observed	Not observed	58.2	0.9

^a^ Calculated as the mass loss of AB in the presence of high-boiling-point amines prior tp the release of any IR-detectable compound.

**Table 3:** Summary of high-boiling-point amines used with their major properties.

Amine	Melting Point (°C)	Boiling Point (°C)	pKa	Amine/AB Acronym
Aniline	−6	184	4.1	aAB
triphenylamine	127	347	3.04	tfaAB
benzene-1,4-diamine	113	267	4.5	panAB
4-methoxyaniline	57	243	5.34	pdaAB

## Data Availability

Data will be provided upon reasonable request to the authors.
